# Activation of GRP78 ATPase suppresses A549 lung cancer cell migration by promoting ITGB4 degradation

**DOI:** 10.1080/19336918.2022.2130415

**Published:** 2022-10-06

**Authors:** Junya Ning, Xiaoling Cui, Nan Li, Na Li, Baoxiang Zhao, Junying Miao, Zhaomin Lin

**Affiliations:** a Shandong Provincial Key Laboratory of Animal Cells and Developmental Biology, School of Life Science, Shandong University, Qingdao, P.R. China; b Key Laboratory of Cellular Physiology at Shanxi Medical University, Ministry of Education, Key Laboratory of Cellular Physiology of Shanxi Province, and the Department of Physiology, Shanxi Medical University, Taiyuan, P.R. China; c Institute of Organic Chemistry, School of Chemistry and Chemical Engineering, Shandong University, Jinan, P.R. China; d Central Research Laboratory, the Second Hospital, Shandong University, Jinan, P.R. China

**Keywords:** Integrin β4, cell migration, glucose-regulated protein 78, hypochlorous acid probe, lung cancer cells

## Abstract

Hypochlorous acid (HOCl) is an essential signal molecule in cancer cells. Activated GRP78 ATPase by a HOCl probe named ZBM-H inhibits lung cancer cell growth. However, the role and underlying mechanism of GRP78 ATPase in lung cancer cell migration have not been established. Here, we reported that activation of GRP78 ATPase by ZBM-H suppressed A549 cell migration and inhibited EMT process. Notably, ZBM-H time-dependently decreased the protein level of integrin β4 (ITGB4) in A549 cells. Combinatorial treatment of 3BDO (an autophagy inhibitor) and ZBM-H partially rescued the protein level of ITGB4. Consistently, 3BDO partially reversed ZBM-H-inhibited cell migration. Furthermore, ZBM-H promoted the interaction between ANXA7 and Hsc70, which participated in the regulation of selective autophagy and degradation of ITGB4.

## Introduction

Lung cancer is one of the most frequently diagnosed cancers and the leading cause of cancer-related deaths worldwide with an estimated 2 million new cases and 1.76 million deaths per year. Non-small-cell lung cancer (NSCLC) accounts for 85% of total diagnoses [1]. Metastasis of lung cancer cells from primary tumors in the lung to distal organs is the major driver of its high mortality rate. Patients with metastatic NSCLC have a poor prognosis [2]. Therefore, finding a potential therapeutic target for NSCLC metastasis is important for clinical treatment. Hypochlorous acid is an essential signal molecule in the regulation of cancer cell fate. Studies have found that hypochlorous acid and hypochlorite play an important role in cell migration [3–5]. However, it is not clear whether endogenous hypochlorous acid is involved in cancer cell migration.

Glucose-regulated protein 78 (GRP78), a member of heat shock protein 70 family, is a multifunctional endoplasmic reticulum chaperone. Expression of cell surface GRP78 (CS-GRP78) increases cancer malignant behavior by augmenting proliferate, invasive and metastatic potential of cancer cells [6]. GRP78 plays an important role in the invasion and metastasis of lung cancer by mediating the interactions between lung cancer and tumor microenvironment, and promoting epithelial–mesenchymal transition [7]. Activated GRP78 ATPase by ZBM-H promotes lung cancer cell apoptosis [8]. However, the role of GRP78 ATPase in lung cancer metastasis remains unclear.

Integrin β4 (ITGB4) is a tumor-associated antigen, which is highly expressed in a variety of malignant tumors and plays a role in promoting tumor metastasis [9,10]. ITGB4 overexpression is associated with venous invasion and decreased overall survival in NSCLC [11]. A recent study showed that NEDD4-dependent ITGB4 ubiquitination degradation effectively inhibited breast cancer cell migration [12]. Besides, autophagy is essential for protein quality control. Our previous study has demonstrated that ZBM-H-induced activation of GRP78 ATPase stimulated autophagy in A549 cells [13]. We wondered whether ZBM-H-induced autophagy participates in regulating the level of ITGB4 and cell migration.

In the present study, we attempted to evaluate the effect of small molecule ZBM-H targeting hypochlorous acid on lung cancer cell migration and to clarify the role and underlying mechanism of activated-GRP78 ATPase by ZBM-H in lung cancer cell migration. Our substantial evidence suggests that activated-GRP78 ATPase by ZBM-H increases selective autophagy and degradation of ITGB4, thereby inhibiting A549 cell migration.

## Materials and methods

### Antibodies

Antibodies for LC3B (2775S) were from Cell Signaling Technology (CST), The United States. β-actin (A5441) antibody was from Sigma Aldrich. ANXA7 (sc-17815) antibody was from Santa Cruz Biotechnology, The United States. Antibodies for ITGB4 (21738-1-AP), Hsc70 (10654-1-AP), E-Cadherin (60335-1-Ig) and Vimentin (60330-1-Ig) were from Proteintech Group, China. Horseradish peroxidase-conjugated secondary antibodies were from Jackson immunoresearch. Secondary antibodies for immunofluorescence were goat anti-rabbit IgG Alexa Fluor-546 (A-11035; Invitrogen) and goat anti-mouse IgG Alexa Fluor-633 (A21126; Invitrogen).

### Cell culture

Human lung cancer cell line A549 cells were grown in RPMI Medium 1640 (Gibco, 3180–022) with 10% fetal bovine serum (FBS; Hyclone, SV30087.02). The cell line was cultured in a humidified incubator at 37°C with 5% CO_2_.

### Wound healing assay

A549 cells were seeded into 6 cm culture plates. After the cell density reaches 60%–70%, a 200 μl pipette tip was used to make scratch wounds on the cell monolayer, and the cells were washed twice with PBS to remove all non-adherent cells, following with medium containing ZBM-H. Then the cells were put in the CO_2_ incubator to continue culturing. Finally, an inverted phase-contrast microscope was used to observe the cell status and collect 0 h and 24 h cell images for statistical analysis.

### Western blot analysis

Western blot was conducted as we described previously. Briefly, protein concentration was assessed by the Bradford assay. Then, 30 μg total protein were applied to SDS-PAGE and transferred to PVDF membranes (Millipore, USA), followed by incubation with primary antibody overnight at 4°C. Then corresponding secondary antibodies was incubated for 1 h at room temperature. Proteins were detected with an enhanced chemiluminescence detection kit (Thermo Fisher, 34,080). Finally, the protein relative quantity was analyzed by Image J software.

### Immunofluorescence Assay

Treated cells were fixed with 4% paraformaldehyde (w/v) for 10 min and then incubated with normal goat serum (1:30) for 30 min and primary antibodies (1:100) overnight at 4°C. Cells were washed with PBS for 3 times, and then incubated with secondary antibodies (1:200). Laser scanning confocal microscopy (Zeiss LSM700, Germany) was used to detect fluorescence.

### Statistical analysis

Data from at least three separate experiments were presented as means ± SE and analyzed by t-test with SPSS 17.0 (SPSS Inc., Chicago, IL, USA). Differences at p < 0.05 were considered as statistically significant.

## Results

### ZBM-H inhibits migration of A549 cells

**Figure 1.**
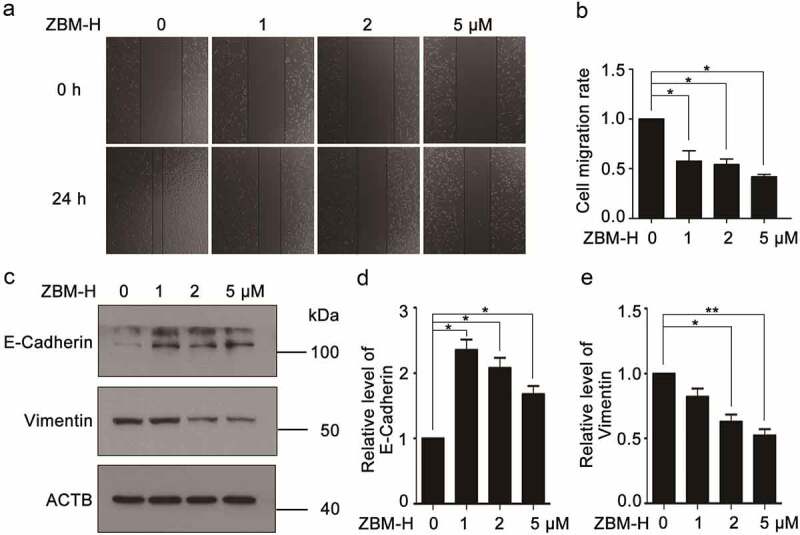
**ZBM-H inhibits the cell migration of A549 cells**. (a-b), A549 cells were scratched and then treated with ZBM-H at indicated concentrations for 24 h. Relative wound closure was quantified by measuring the width of the wounds. (c-e), Western blot analysis of E-Cadherin and Vimentin in A549 cells were treated with ZBM-H for 24 h. Data are presented as the mean ± SEM, *p < 0.05, **p < 0.01, n = 3.

Migration and infiltration function as crucial factors in tumor progression [14]. To explore whether small molecule ZBM-H affected migration in A549 cells, we conducted wound healing assay. Data revealed that ZBM-H effectively suppressed A549 cells migration (Figure 1a, and b).

Epithelial–mesenchymal transition (EMT) is an essential biological process for cells to acquire the ability of migration [15]. Then, we analyzed the effect of ZBM-H on the EMT process of A549 cells. The results indicated that ZBM-H inhibited the EMT process of A549 cells as evidenced by increased epithelial marker E-Cadherin and decreased mesenchymal marker Vimentin (Figure 1c–e).

### ZBM-H negatively regulates the protein level of ITGB4 by inducing autophagy

**Figure 2.**
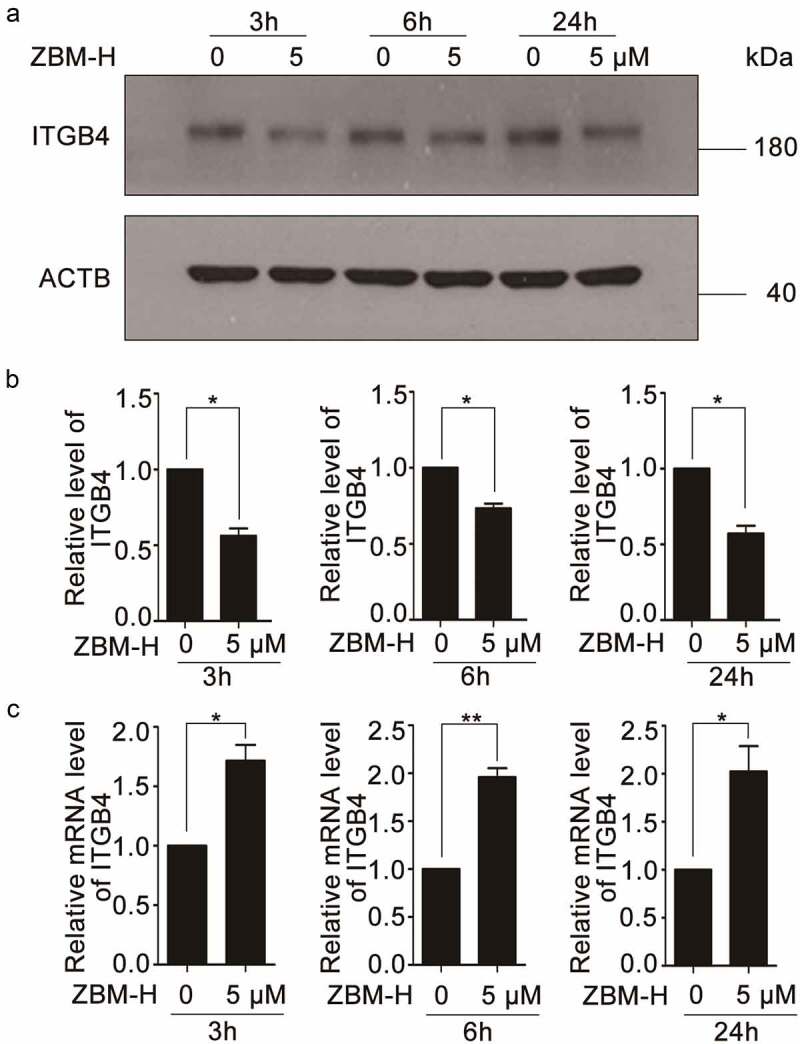
**ZBM-H reduces the protein level of ITGB4 while increases ITGB4 mRNA level**. (a-b), Western blot analysis of ITGB4 in A549 cells treated with ZBM-H (5 μM) for 3 h, 6 h and 24 h. (c), A549 cells were treated with ZBM-H (5 μM) and then the mRNA level of ITGB4 was detected by qPCR. Data are presented as the mean ± SEM, *p < 0.05, **p < 0.01, n = 3.

Emerging evidence suggests that high level of ITGB4 is closely related to cell migration and invasion [9]. Western blot analysis showed that ZBM-H clearly reduced the protein level of ITGB4 in a time-dependent manner (Figure 2a, and b). However, qPCR analysis revealed that ITGB4 mRNA level increased in A549 cells treated with ZBM-H (Figure 2c). In addition, the effects of ZBM-H on the other integrins (integrin β1, integrin β3, and integrin β6) involved in cell migration were determined. Western blot analysis showed that ZBM-H had no significant effect on integrin β1 (ITGB1) and integrin β3 (ITGB3) (Supplementary Figure S1-S2). The protein level of integrin β6 (ITGB6) was not significantly altered after ZBM-H treatment, except for a slight decrease at 6 h (Supplementary Figure S3). The qPCR analysis indicated that the mRNA levels of ITGB1 and ITGB6 significantly decreased with ZBM-H treatment for 3 h (Supplementary Figure S1 and Figure S3). These results suggested that ZBM-H inhibited cell migration by decreasing the protein level of ITGB4.

**Figure 3.**
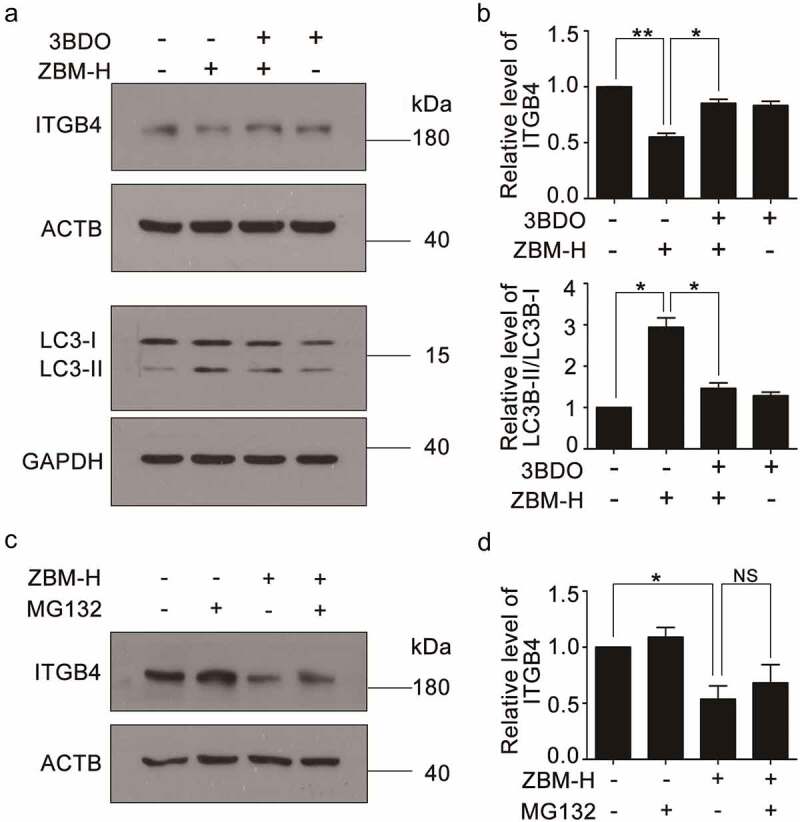
**ZBM-H negatively regulates the protein level of ITGB4 through autophagy instead of the proteasome degradation pathway**. (a-b), Western blot analysis of ITGB4 and LC3B-II/LC3B-I in A549 cells treated with ZBM-H (5 μM) and 3BDO (1 μM) for 24 h. (c-d), Western blot analysis of ITGB4 in A549 cells treated with ZBM-H (5 μM) and MG132. Data are presented as the mean ± SEM, NS p > 0.05, *p < 0.05, **p < 0.01, n = 3.

Autophagy, especially chaperone-mediated autophagy, plays an important role in protein quality control [16]. Given that ZBM-H promoted autophagy in A549 cells, we wondered whether autophagy was involved in the regulation of ITGB4 protein level. Combinatorial treatment of 3-benzyl-5-((2-nitrophenoxy) methyl)-dihydrofuran-2(3 H)-one (3BDO), an activator of mechanistic target of rapamycin kinase complex 1 (mTORC1), and ZBM-H surely inhibited autophagy and partially rescue ITGB4 protein levels (Figure 3a, and b). In addition, the proteasome degradation pathway is also crucial for the regulation of protein quality [17]. Studies have shown that ITGB4 was degraded through the ubiquitin-proteasome pathway [12,18]. Therefore, we treated A549 cells with ZBM-H and proteasome inhibitors MG132. The results showed that MG132 failed to rescue ITGB4 protein level significantly (Figure 3c and 3d). These findings indicated that ZBM-H negatively regulates the protein level of ITGB4 by inducing autophagy.

### ZBM-H inhibits cell migration by promoting autophagy

**Figure 4:**
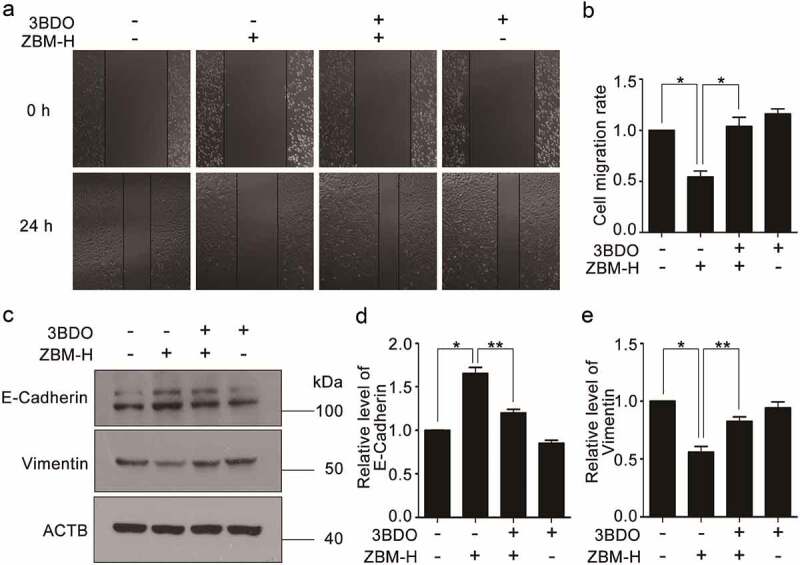
**3BDO reverses the inhibitory effect of ZBM-H on cell migration. (a-b)**, A549 cells were scratched and then treated with ZBM-H (5 μM) and 3BDO (1 μM) for 24 h. Relative wound closure was quantified by measuring the width of the wounds. **(c-e)**, Western blot analysis of E-Cadherin and Vimentin in A549 cells treated with ZBM-H (5 μM) and 3BDO (1 μM) for 24 h. Data are presented as the mean ± SEM, *p < 0.05, **p < 0.01, n = 3.

To further get an in-depth understanding of the mechanism by which ZBM-H inhibited cell migration, we firstly conducted wound healing assay by using autophagy inhibitor 3BDO. Data showed that 3BDO surely attenuated ZBM-H-inhibited cell migration (Figure 4a, and b). In addition, western blot analysis showed that 3BDO and ZBM-H co-treatment inhibited the protein level of the epithelial marker E-Cadherin and increased the protein level of the mesenchymal marker Vimentin (Figure 4c, –e). These findings further indicated that ZBM-H inhibits EMT and cell migration by promoting autophagy.

**3.4 ZBM-H regulates the selective autophagy and degradation of ITGB4 by promoting the interaction between ANXA7 and Hsc70**

**Figure 5.**
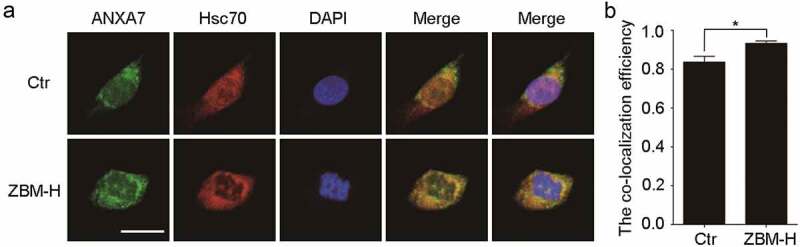
**Immunofluorescence (IF) assay of the co-localization analysis of ANXA7 and Hsc70**. (a-b), A549 cells were treated with ZBM-H (5 μM) for 24 h, and the co-localization of ANXA7 and Hsc70 was analyzed and visualized by confocal immunofluorescence microscopy. Scale bar: 20 μm, *n = 3.*

It has been reported that Hsc70 is a key factor in mediating selective autophagy [16]. In our previous work, we found that ANXA7 was able to interact with ITGB4 [19], so we speculate that ZBM-H may affect the interaction between ANXA7 and Hsc70 by regulating the activity of ANXA7, and ultimately induce ITGB4 protein degradation through selective autophagy. Immunofluorescence analysis showed that ZBM-H changed the cell distribution of Hsc70 and promoted the co-localization of ANXA7 and Hsc70 (Figure 5a, and b). This finding indicated that ZBM-H promotes the interaction between ANXA7 and Hsc70, thereby regulating the selective autophagy and degradation of ITGB4.

## Discussion

ITGB4 protein level is positively correlated with cell migration [9]. Our previous study elucidated that hypochlorous acid probe named ZBM-H was able to enhance the activity of GRP78 [13]. In this study, we found that activated GRP78 by ZBM-H surely inhibited cell migration. At the same time, the protein level of ITGB4 decreased, but ITGB4 mRNA level increased in cells treated with ZBM-H. Therefore, we speculated that the degradation of ITGB4 protein may increase. Autophagy and ubiquitin-proteasome pathway are two major pathways for protein quality control [16,20]. In the further study, we found that inhibiting autophagy by 3BDO partially rescue ITGB4 protein level while proteasome inhibitors MG132 failed to rescue ITGB4 protein level. In addition, 3BDO reversed ZBM-H-inhibited EMT and cell migration, which indicated that ZBM-H regulated ITGB4 protein level by stimulating autophagy instead of ubiquitin-proteasome pathway, thereby inhibiting cell migration. It has been reported ubiquitination-mediated proteasome and lysosomal degradation pathways as well as protease cleavage degradation pathways were involved in the regulation of ITGB4 degradation [12,18,21]. However, our study provides new evidence for the autophagic degradation of ITGB4.

Studies have found that under certain conditions, cells activate chaperone-mediated autophagy (CMA) and promote the selective degradation of proteins to maintain their energy requirements. Normal proteins and oxidative stress-induced damaged proteins can anchor to the lysosome membrane with the assistance of Hsc70 and enter the lysosome for degradation, thereby achieving protein quality control and downstream signal pathway regulation. Normally, Hsc70 directly binds to the cytoplasmic protein that needs to be degraded, and then recognizes the receptor protein LAMP2A to promote the selective degradation of the substrate protein [16,22–24]. It is generally believed that damaged or abnormally synthesized proteins containing the KFERQ motif are the main substrates of CMA. However, a recent study revealed that normal proteins can also be substrates of CMA during specific cellular processes [25].

Annexin A7 (ANXA7) is a member of annexin superfamily. As a Ca^2+^-dependent, phospholipid-binding protein, ANXA7 performs a variety of functions in cells, including facilitating vesicle transport and membrane fusion via its GTPase activity [26,27]. Our previous study determined that small chemical molecule ZBM-H was able to increase the activity of ANXA7 by promoting the interaction between GRP78 and ANXA7 [8]. Given that ANXA7 can translocate to the lysosome, we hypothesized that ANXA7 may be involved in selective autophagy. Our current study revealed that ZBM-H promoted the interaction between ANXA7 and Hsc70. In additon, ANXA7 was able to interact with ITGB4 [19]. Therefore, we speculate that ANXA7 may play two roles in the selective degradation of ITGB4. One possibility is that ANXA7 can act as a scaffold protein to promote the formation of ITGB4-ANXA7-Hsc70 complex, and the other is that ANXA7 may help Hsc70 anchor to the lysosome to recognize the relevant receptor. This hypothesis will be verified in our following research.

**Figure 6.**
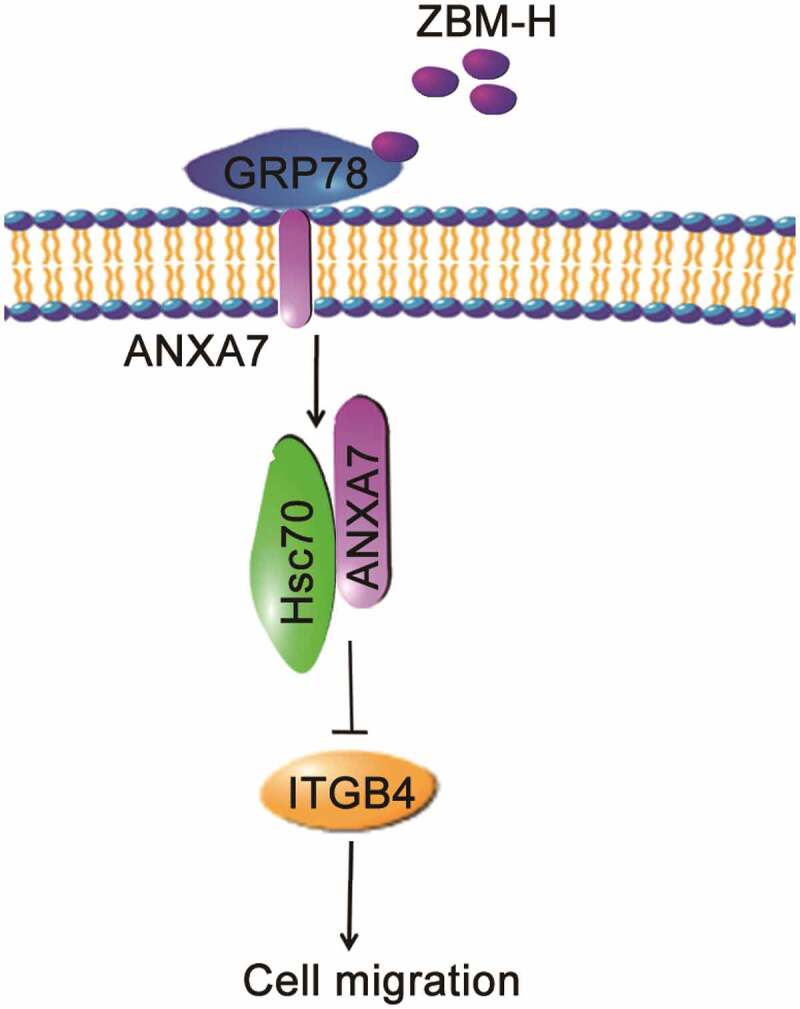
Schematic of activation of GRP78 ATPase by ZBM-H suppresses A549 lung cancer cell migration.

In conclusion, this study revealed that the small chemical molecule ZBM-H targeting HOCl enhanced the interaction between ANXA7 and Hsc70, which further participated in ITGB4 degradation though selective autophagy, thereby inhibiting A549 cell migration (Figure 6).

## Supplementary Material

Supplemental Material

## Data Availability

The authors confirm that the data supporting the findings of this study are available within the article.
