# Supplementary material for: Activation of GRP78 ATPase suppresses A549 lung cancer cell migration by promoting ITGB4 degradation
Source: Cell Adh Migr. 2022 Oct 6;16(1):107–14. doi: 10.1080/19336918.2022.2130415 (PMC9542429; doi:10.1080/19336918.2022.2130415)
Supplement: Supplemental Material [file KCAM_A_2130415_SM5596.docx]

**Activation of GRP78 ATPase suppresses A549 lung cancer cell migration by promoting ITGB4 degradation**

Junya Ning^a，b^, Xiaoling Cui^a^, Nan Li ^a^, Na Li ^a^, Baoxiang Zhao^c^, Junying Miao^a *^ Zhaomin Lin^d *^

*^a^ Shandong Provincial Key Laboratory of Animal Cells and Developmental Biology, School of Life Science, Shandong University, Qingdao 266237, P.R. China*

*^b^ Key Laboratory of Cellular Physiology at Shanxi Medical University, Ministry of Education, Key Laboratory of Cellular Physiology of Shanxi Province, and the Department of Physiology, Shanxi Medical University, Taiyuan 030001, P.R. China*

*^c^ Institute of Organic Chemistry, School of Chemistry and Chemical Engineering, Shandong University, Jinan 250100, P.R. China*

*^d^ Central Research Laboratory, the Second Hospital, Shandong University, Jinan,*

*250033, P.R. China*

*Correspondence to: Prof. Junying Miao and Dr. Zhaomin Lin, Shandong Provincial Key Laboratory of Animal Cells and Developmental Biology, School of Life Science, Shandong University, Qingdao 266237, P.R. China,

E-mail address: [miaojy@sdu.edu.cn](mailto:miaojy@sdu.edu.cn) and 201462016218@sdu.edu.cn


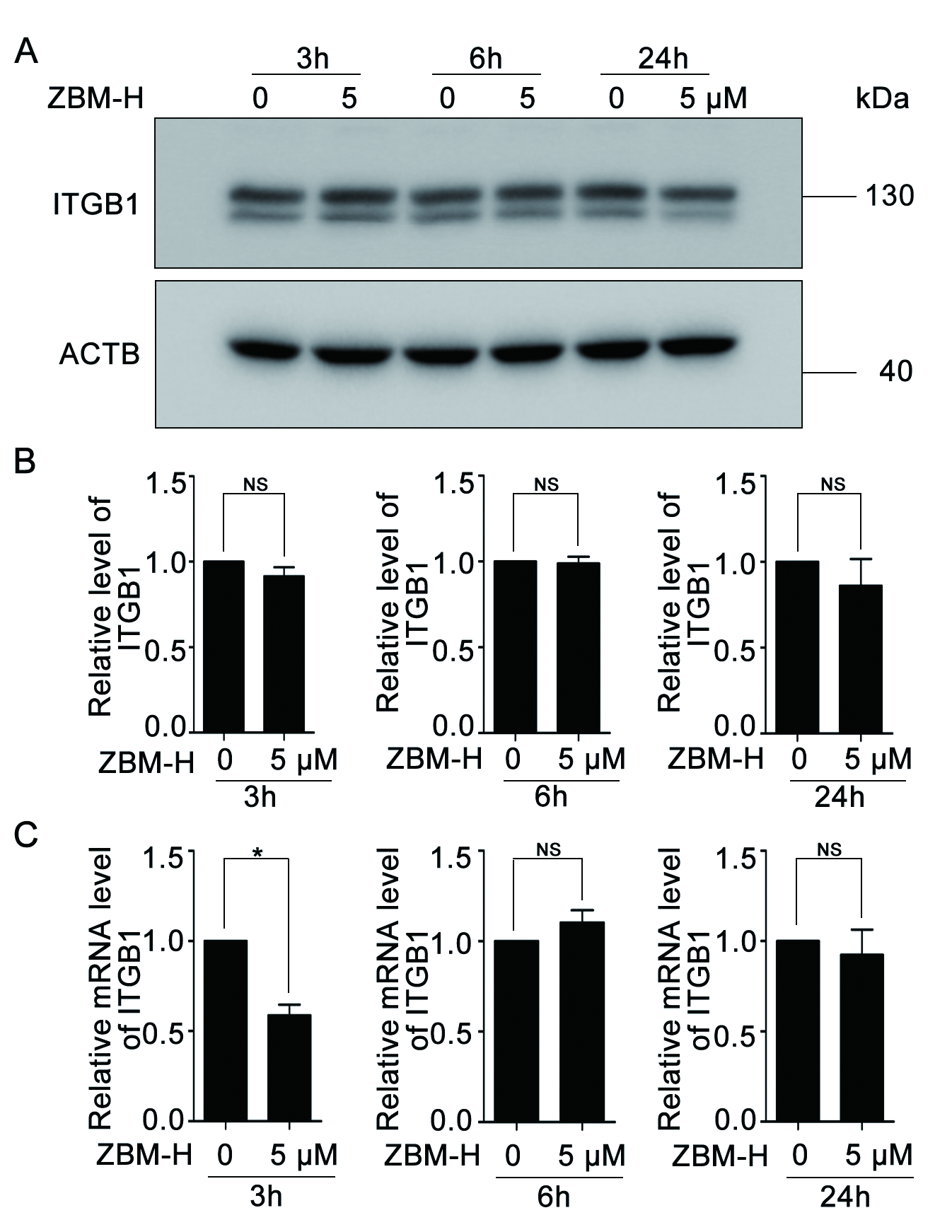


**Supplementary Figure S1 The effect of ZBM-H on the protein and mRNA level of integrin β1 (ITGB1). (A-B)**, Western blot analysis of ITGB1 in A549 cells treated with ZBM-H (5 μM) for 3h, 6h and 24 h. **(C)**, A549 cells were treated with ZBM-H (5 μM) and then the mRNA level of ITGB1 was detected by qPCR. Data are presented as the mean ± SEM, **p<0.05, NS p>0.05, n=3.*


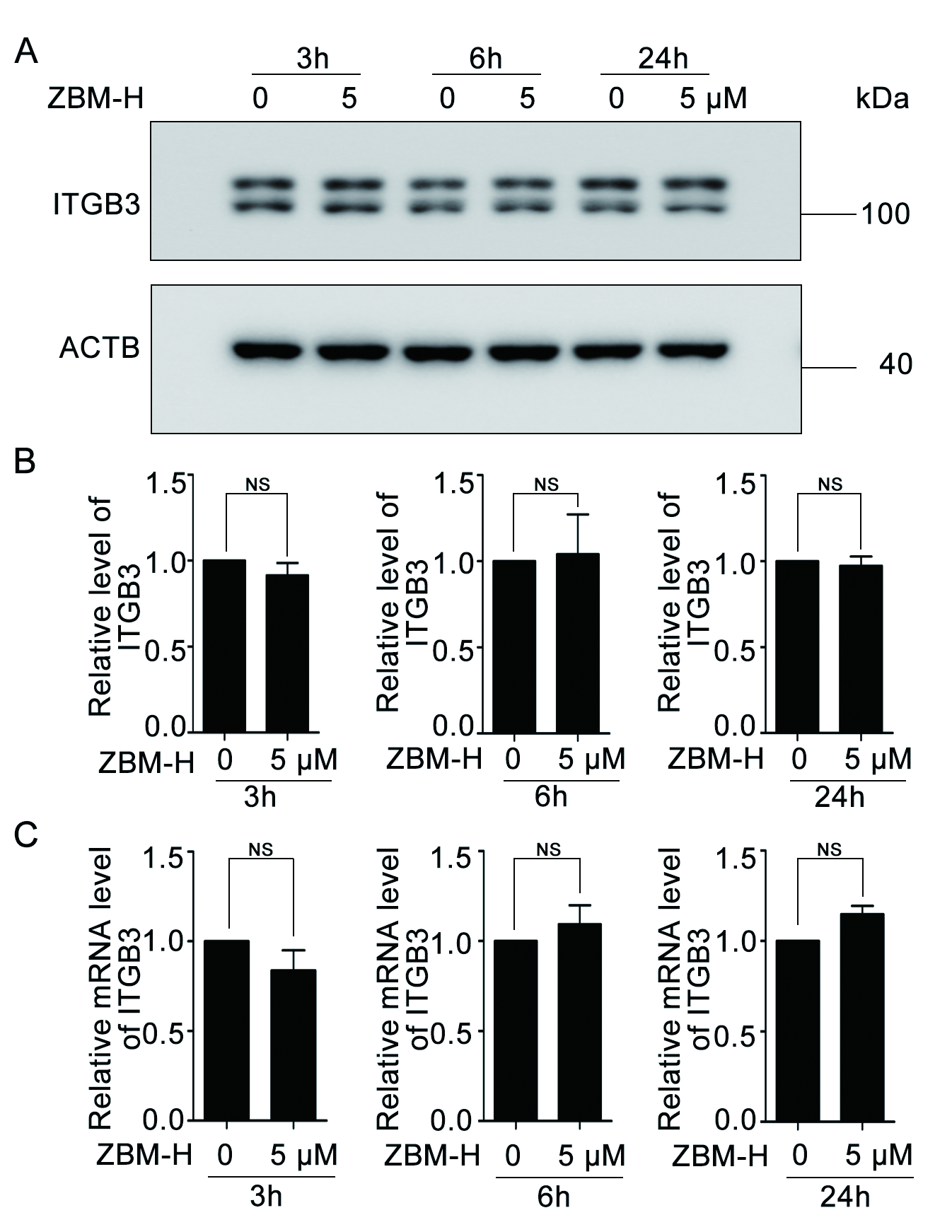


**Supplementary Figure S2 The effect of ZBM-H on the protein and mRNA level of integrin β3 (ITGB3). (A-B)**, Western blot analysis of ITGB3 in A549 cells treated with ZBM-H (5 μM) for 3h, 6h and 24 h. **(C)**, A549 cells were treated with ZBM-H (5 μM) and then the mRNA level of ITGB3 was detected by qPCR. Data are presented as the mean ± SEM, *NS p>0.05, n=3.*


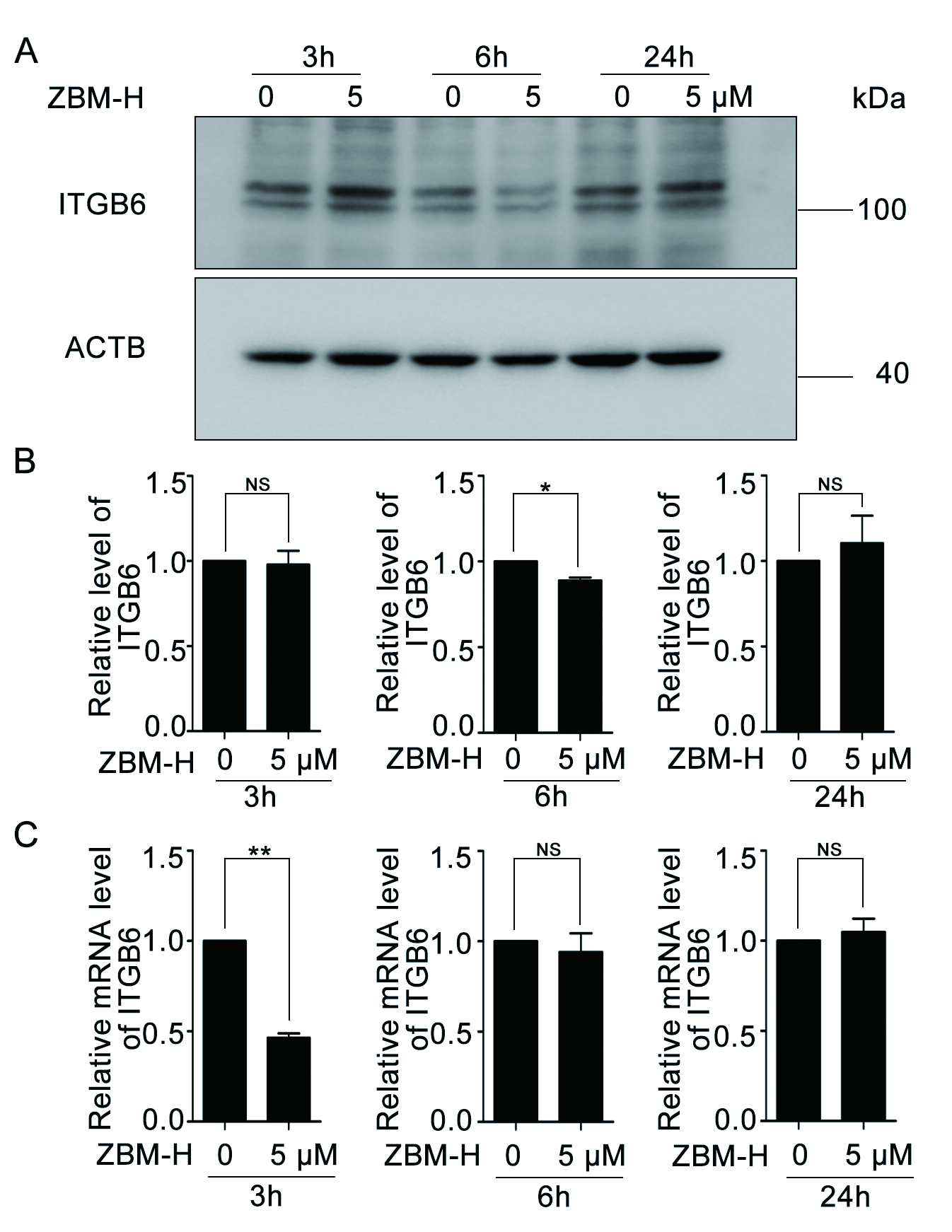


**Supplementary Figure S3 The effect of ZBM-H on the protein and mRNA level of integrin β6 (ITGB6). (A-B)**, Western blot analysis of ITGB6 in A549 cells treated with ZBM-H (5 μM) for 3h, 6h and 24 h. **(C)**, A549 cells were treated with ZBM-H (5 μM) and then the mRNA level of ITGB6 was detected by qPCR. Data are presented as the mean ± SEM, **p<0.05, **p<0.01, NS p>0.05, n=3.*
